# The impact of non-native trees on galling and herbivory in New York City across space and time

**DOI:** 10.1007/s00442-026-05951-0

**Published:** 2026-09-04

**Authors:** Liam D. Engel, Adanna Smith, Irma Cardenas, Dunya Abdulkarem, Claudia C. Vasquez, Evon Hekkala, Emily M. Herstoff, Michael Tessler

**Affiliations:** 1 Department of Biological Sciences, Fordham University, Bronx, NY 10458 USA; 2 Department of Biology, Medgar Evers College, Brooklyn, NY 11225 USA; 3 Department of Biology, St. Francis College, Brooklyn, NY 11201 USA; 4 Institute for Comparative Genomics, American Museum of Natural History, New York, NY 10024 USA; 5 Division of Invertebrate Zoology, American Museum of Natural History, New York, NY 10024 USA; 6 Center for Biodiversity and Evolution, New York Botanical Garden, Bronx, NY 10458 USA; 7 Graduate Center, City University of New York, New York, NY 10016 USA

**Keywords:** Arthropods, Community science, Congeneric pairs, Galls, Generalists, Herbarium specimens, Herbivory, Specialists

## Abstract

Cities and suburbs frequently plant native and non-native trees as foundation species, with non-natives cultivated in these areas for centuries while remaining non-invasive. Although previous research has found that native trees often host more arthropods, studies have not simultaneously looked across space and time to determine the consistency of tree origin on urban arthropods. We combined varied methods across spatial and temporal scales in New York City to test if native tree leaves consistently have more insect and mite interactions than long-established non-native trees, predicting stronger effect sizes for specialists (galling arthropods) than generalists (herbivory). We examined (1) congeneric species pairs, controlled for growing conditions and stoichiometry in an arboretum, (2) diverse oaks at a botanical garden, (3) community science records across Brooklyn, and (4) herbarium specimens from 1883 through present across the city. Across spatiotemporal scales, we found consistent results. Specialist interactions were striking: contemporary native trees supported numerous galling species, while only one congeneric non-native species hosted any galls. For generalists, contemporary native trees had equivalent to slightly greater herbivory. Over the last century, herbarium records showed that herbivory increased on non-native trees to nearly the level of natives, whereas native trees increased in gall abundance while non-native trees remained rarely galled. Our results demonstrate the impact of tree origin on tree-arthropod interactions in a real-world urban setting, with far fewer galls even when non-native tree species have been cultivated locally for centuries. Our findings will help city planners and property owners confidently choose native trees to promote arthropod biodiversity.

**Supplementary Information:**

The online version contains supplementary material available at https://doi.org/10.1007/s00442-026-05951-0.

## Introduction

Non-native plants have rapidly spread across the globe (Vitousek et al. 1997), partly due to landscaped environments (Tallamy et al. 2021). In the United States, European colonization led to the introduction of non-native trees from the 1500s onward (Lewis 1976), with many plantings in the 1800s (National Research Council 2002). Centuries later, non-natives still often comprise > 40% of all individual trees planted in urban and suburban areas (Schlaepfer et al. 2020; Doroski et al. 2020; Liu and Slik 2022). Native tree species generally support greater faunal biodiversity in urban areas (Tartaglia and Aronson 2024), but non-native tree species may still support certain taxa (Chalker-Scott 2015; Gray and Van Heezik 2016; Shackleton 2016; Berthon et al. 2021). However, many studies have been limited in terms of geographic and time scales (Laux et al. 2022). Here we examine how arthropods (primarily insects), with two levels of host specialization, interact with commonly-planted native and non-native trees across multiple spatial and temporal scales within New York City, including changes over the last two centuries.

Studies comparing arthropod enemies on native and non-native trees have found striking differences between specialists (e.g. gall formers) and generalists (e.g. herbivores) (Burghardt and Tallamy 2013; Kirichenko et al. 2013). The enemy release hypothesis posits that non-native species will be released from natural enemies upon introduction to new areas, with particular release from specialized, co-evolved species (Keane and Crawley 2002). Gall-forming arthropods cause plant tissues to produce specialized growths providing nutrients and protection (Harris and Pitzschke 2020), and these interactions are highly conserved (Lopez-Vaamonde et al. 2001; Ronquist and Liljeblad 2001; McLeish et al. 2007; Stone et al. 2009; Warren et al. 2021). Host plants moved to new habitats rarely transport their gall-forming symbionts (Warren et al. 2021), although there are exceptions (e.g. Chestnut gallwasp; Csóka et al. 2017). While leaf herbivory (i.e. folivory) can be specialized (e.g. monarch caterpillars and milkweeds) (Agrawal 2005), it is often a more generalized interaction between arthropods and plant hosts compared to galling (Cates 1981; Loxdale and Harvey 2016). Although many studies found lower levels of leaf herbivory on non-native plants (Agrawal et al. 2005; Hill and Kotanen 2009; Matter et al. 2012), a meta-analysis of the enemy release hypothesis found similar levels of insect herbivory on native and non-native plants (Meijer et al. 2016). In contrast, congeneric studies on woody plants have found greater herbivory on native species (Pearse and Hipp 2009; Burghardt and Tallamy 2013; Clem and Held 2018). Ultimately, it is important that comparisons with non-natives (taxa or guilds) be conducted using congeneric species across multiple spatial and temporal scales to reduce phylogenetic distance and directly test the importance of plant origin, but few studies have utilized this approach.

Studies of arthropods on native and non-native plants have ranged from arboreta comparisons to common garden experiments, including in urbanized sites (Matter et al. 2012; Burghardt and Tallamy 2013; Lerch et al. 2024). However, studies have not been done at differing urban scales (e.g. from a site through a whole city). Studying multiple urban scales is critical, as individual sites can vary dramatically in land management, human population density, and local ecological communities. Examinations across time are possibly even more critical, as they give a trajectory of plant-arthropod interactions (e.g. have these relationships plateaued or are they still likely to increase). Prior results have similar trends — native plants support more insects — but noteworthy differences exist (Hawkes 2007; Brändle et al. 2008; Meijer et al. 2016). In one study, hundreds of years after introduction, contemporary woody non-natives hosted fewer insect specialists (Brändle et al. 2008). In contrast, a meta-analysis found enemy attacks on non-native plants mirrored native levels in as quickly as 50 years, depending on taxa (Hawkes 2007). In part, differences could be because studies often infer past relationships rather than directly examine them (Hawkes 2007; Brändle et al. 2008; Schulte et al. 2025). However, herbaria can let us explore over two centuries of plant-insect interactions (Daru and Zhigila 2025). One herbarium study found herbivory on US native species increased over > 100 years, except in urban areas (Meineke et al. 2019). A Norwegian herbarium study found leaf herbivory did not change through time for congeneric native and non-native plants (Ivison et al. 2023).

Ecological stoichiometry (e.g. C:N:P) can drive consumer food choice (Sterner and Elser 2002), and congeneric plant species are expected to have similar stoichiometry (Blomberg et al. 2003; Rothwell and Holeski 2020). We are unaware of any studies that examine chemical traits of congeneric native and non-native plants as a mechanism to explain differences in biodiversity hosted by plants. Our recent work in Green-Wood Cemetery, Brooklyn (New York City) controlled for this, and found that congeneric pairs of native and non-native trees are generally similar chemically as compared to other genera (Herstoff et al. 2025). Like most cities, New York City is biologically heterogeneous with significant land use changes over the last centuries. New York City’s biodiversity and ecology remain unevenly studied in the scientific literature (Felson et al. 2014; Herstoff et al. 2024), but presents useful study characteristics. For example, non-native and native plant congeners are often grown close together (Street Tree Planting Standards for New York City, 2016), such as in New York City’s long-established planted greenspaces, including arboreta like Green-Wood Cemetery (founded 1838) (Herstoff et al. 2025; Rae 2021) and gardens like Brooklyn Botanic Garden (founded 1910). Furthermore, many records have been gathered in New York City by both professional herbarium collections and community science efforts (iNaturalist has > 270,000 observations for Brooklyn alone), making it possible to compare field-based research to known contemporary and historic records.

In our analyses, we explore predictions about specialists and generalists relating to the enemy release hypothesis. We compare common native and non-native tree species in New York City across temporal and spatial scales, as differences likely exist despite non-natives having been planted for hundreds of years. We test five hypotheses. *H*_*1*_: Native tree leaves will have greater mean percent herbivory and gall abundance than non-native congeners. *H*_*2*_: The effect size will be higher for arthropod interactions that are specialized (galls) versus generalized (herbivory). *H*_*3*_: Results will be consistent across spatial scales (single sites, single borough, and whole city), despite historical and geological variation. *H*_*4*_: Herbivory will increase over time on non-native species, but urbanization over time may temper this (Meineke et al. 2019). *H*_*5*_: Gall abundance on non-native hosts will be consistently low over time.

## Methods

### Overview

**Fig. 1:**
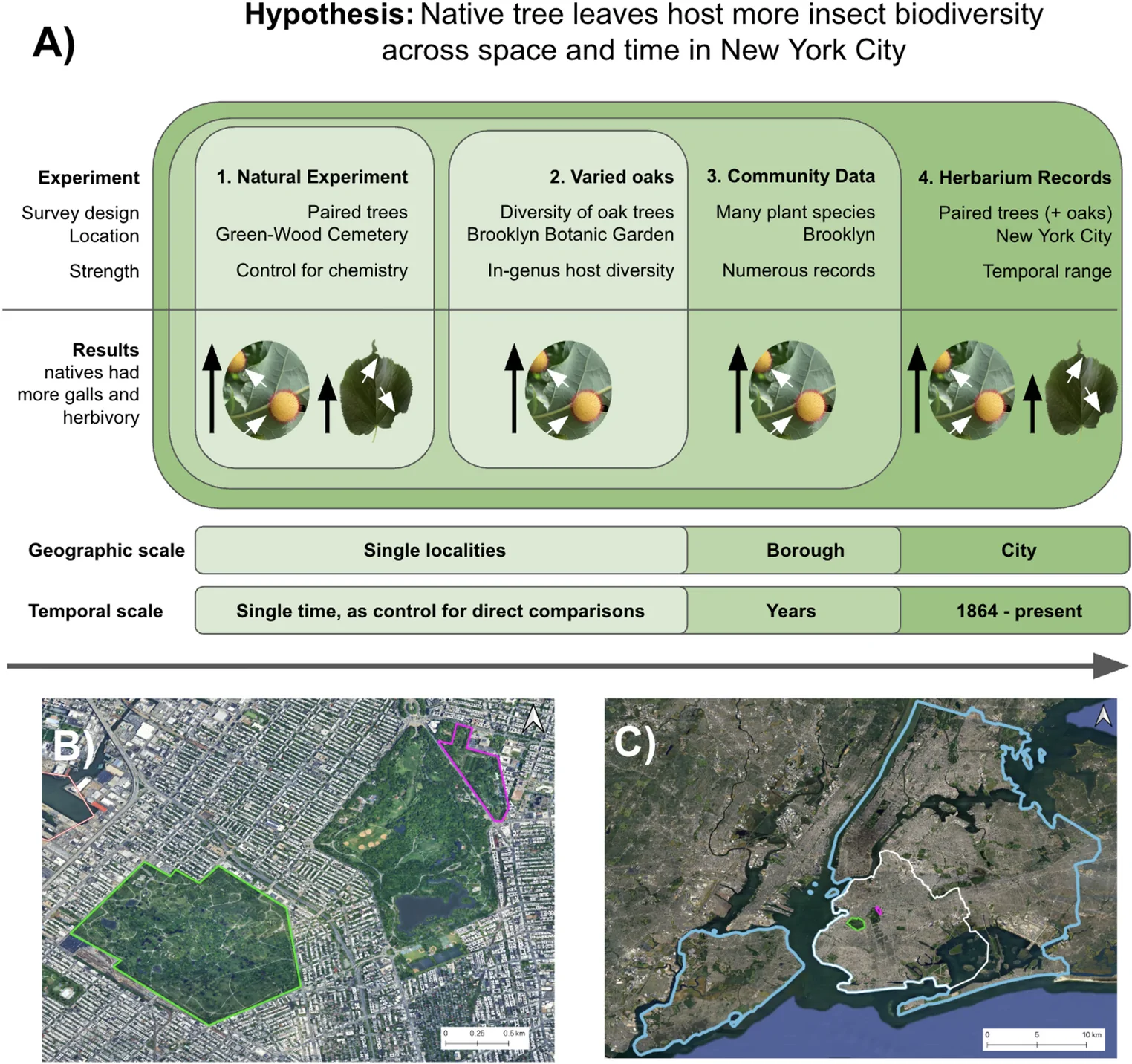
(**A**) A graphical abstract of our experimental design, study sites, and results. Four substudies tested our hypotheses. The images in the results represent our examination of gall-forming arthropods (specialized leaf parasites, depicted as a circular image of yellow *Acraspis erinacei* on a native *Quercus alba* leaf) and leaf herbivory (generalized feeding by insects, depicted as chewmarks on a native *Tilia americana* leaf). The dark, vertically oriented arrows indicate the effect size of our findings, with galls being considerably more abundant and diverse on native species, and herbivory being equal to or greater on nearly all natives as compared to non-natives. Experiments 1 and 4 examined galls and herbivory, whereas experiments 2 and 3 focused on galls only. The maps show (**B**) our study sites in Brooklyn, and (**C**) our greater sampling areas in New York City, New York. The maps include live tree sampling in (1) Green-Wood Cemetery (green) and (2) Brooklyn Botanic Garden (pink); and digital image records in (3) Brooklyn (white; iNaturalist records), and (4) New York City (light blue; New York Botanical Garden herbarium specimens)

To test our hypotheses, we examined the prevalence of specialists (gall-forming arthropods) and generalists (leaf herbivory) on native and non-native trees across geographic and temporal scales. An overview of the four substudy designs is presented in Fig. 1, with sampling locations marked in Supplemental Fig. 3. The spatial range of the substudies spanned localized areas in Brooklyn, the borough of Brooklyn (Kings County), and all five boroughs of New York City. The temporal range of the substudies spanned collections at single time periods, through the last decade, through more than a century of observations.

#### Natural experiment with congeneric pairs: Green-Wood Cemetery, Brooklyn

This substudy focused on comparing congeneric native and non-native trees grown in highly similar conditions at a constrained time point and location (summer 2024 in Green-Wood Cemetery; Fig. 1, Supplemental Fig. 3). We consider this a natural experiment, as it is a naturally occurring common garden — uniform conditions over decades of growing, coupled with the dispersed plantings of congeneric native and non-native species.

Green-Wood Cemetery (Brooklyn, NY; 40°39′09″ N, 73°59′28″ W) is a National Historical Landmark and an accredited level III arboretum in a high-density urban location. Green-Wood Cemetery has 478 acres, which is covered by a diversity of plantings. The most common planting arrangement, which we focus on here, is specimen trees (crowns sometimes abutting) surrounded by turf grass (lawns). The trees are maintained uniformly, with little maintenance after the first year except for removal of damaged limbs as needed (S. Evans, *pers. comm.*). We have separately analyzed soil chemistry, and found similar soils throughout the site (e.g. soils where natives and non-natives are grown were statistically indistinguishable) (Herstoff et al. 2025). Planted trees include many congeneric species that are native (North American) and non-native (usually Asian or European).

We used Green-Wood Cemetery’s arboretum database to obtain information on our focal trees, including accession number, species, cultivar, condition, diameter at breast height (DBH), location, and year planted. We randomly selected ten trees of each species for this substudy, excluding trees that were grown in inaccessible areas, had inaccessible branches, or were planted recently (i.e. last five years).

We examined galling and herbivory on five native and non-native congeneric species pairs in summer 2024 (Table 1), focusing on maples (*Acer*), cherries (*Prunus*), oaks (*Quercus*; two congeneric pairs), and lindens (*Tilia*). These trees were selected because they are commonly planted in suburban and urban areas, including within New York City (Street Tree Planting Standards for New York City, 2016; Nowak et al. 2018), and because many individuals of these trees have been planted in Green-Wood Cemetery. Most tree species were surveyed July 5–10, with one pair of oaks (*Q. palustris* and *Q. acutissima*) surveyed on July 30 and cherries surveyed on August 23. Two congeneric conifer pairs were also surveyed (*Picea abies* and *Picea glauca*; *Pinus strobus* and *Pinus wallichiana*), but herbivory was minimal and galls were not found; thus these conifers were excluded from the rest of the study.

**Table 1:** Congeneric tree species pairs surveyed for galls and herbivory at Green-Wood Cemetery during summer 2024. *Quercus palustris and *Q. acutissima* were only surveyed for galls. Two congeneric conifer pairs (*Picea* species and *Pinus* species) were also examined but did not display clear signs of herbivory or galls, and were thus excluded from downstream comparisons

Genus	Species	Native or year introduced	Source
*Acer* (maple)	*A. rubrum*, red maple	Native	
*Acer* (maple)	*A. platanoides*, Norway maple	1756	https://www.nrs.fs.usda.gov/pubs/jrnl/1990/ne_1990_nowak_003.pdf
*Prunus* (cherry)	*P. serotina*, black cherry	Native	
*Prunus* (cherry)	*P. serrulata*, East Asian cherry	1846	https://www.nal.usda.gov/exhibits/speccoll/files/original/75c20660ac8f15b9da4c6ca02d9db606.pdf
*Quercus* (oak)	*Q. alba*, white oak	Native	
*Quercus* (oak)	*Q. palustris*, pin oak*	Native	
*Quercus* (oak)	*Q. robur*, English oak	1600s	https://plants.ces.ncsu.edu/plants/quercus-robur/
*Quercus* (oak)	*Q. acutissima*, sawtooth oak*	1862	https://www.srs.fs.usda.gov/pubs/rn/rn_so386.pdf
*Tilia* (linden)	*T. americana*, basswood	Native	
*Tilia* (linden)	*T. cordata*, little leaf linden	Early 1800s	https://www.nps.gov/articles/000/long-littleleaf-linden-plant-story.htm

Gall presence was determined for the randomly-selected ten trees of each species with a four minute survey walking around the entirety of the tree. This was done as the galls tended to have patchy distributions and low abundances; in our preliminary work we found four minutes to be a good balance for finding most galls while being able to cover sites within a constrained number of days. The only exception to this was our survey of lindens (*Tilia)*, where galls were numerous; here we went to north (0° N) and south (180° N) and counted 50 of the nearest accessible leaves at breast height, resulting in a percentage of galled leaves out of 100 for each tree. Galls were identified to species using iNaturalist, consultations with two gall experts (Matthew Wills and Adam Kranz), and the website gallformers.org. Galling arthropods have been documented regularly at Green-Wood Cemetery on iNaturalist and were also used for reference (e.g. >15 gall mite species and > 45 gall wasp species).

Herbivory was documented in the field for the ten randomly selected trees of each species, with photos taken for later measurement. Each photo was taken of a single leaf placed on a white sheet of paper with direct sunlight to minimize shadows, with the leaf taking up most of the photo. The percentage of leaf herbivory was recorded for 10 leaves of each tree split evenly across two cardinal directions: north (0° N) and south (180° N). We selected leaves at approximately breast height, and only the five leaves closest to the stem apex at each cardinal direction were counted. When leaves were unavailable at these heights or directions, the nearest leaves were sampled. Percentage herbivory was calculated from leaf photos by a single surveyor (Engel) after being trained using the ZAX Herbivory Trainer (Xirocostas et al. 2022). Percentages for the ten leaves were averaged to obtain an average leaf herbivory percentage for each of the 10 trees per species, which was used in subsequent statistical analysis.

To determine if galling and herbivory were influenced by host tree baseline stoichiometry, we analyzed leaf tissue for its carbon (C), nitrogen (N), and phosphorus (P) content. We selected these elements because they are key drivers of plant-insect interactions (Sterner and Elser 2002). These tree stoichiometric variables (along with soil chemistry) were reported in one of our prior studies (Herstoff et al. 2025). To briefly recap the methods we used, for four of the initial five genera sampled (i.e. all but *Prunus*), we collected ten leaves or an equivalent mass of needles for six trees of each tree species on 1-Aug-2024. Leaves/needles were selected north (0° N) and south (180° N) from each tree at breast height. If these directions were inaccessible, the closest area(s) were utilized. Plant material was dried overnight and shipped to Dairy One Forage Laboratory for chemical analysis. Our prior study found no significant differences in soil chemistry between native and non-native species; thus we do not include this variable in these analyses (Herstoff et al. 2025).

#### Comparing varied oaks: Brooklyn Botanic Garden, Brooklyn

Because oak trees are of particular ecological importance in the US (Tallamy 2021), have numerous gall-forming arthropod species in Brooklyn (as seen in iNaturalist records), and are diverse themselves (Nixon, 2006), we conducted a focused sampling effort for oaks at Brooklyn Botanic Garden (40°40′12″N, 73°57′45″W). This substudy was conducted at a constrained time point and location (19-20-Sep-2024, in Brooklyn Botanic Garden; Fig. 1, Supplemental Fig. 3).

Brooklyn Botanic Garden is a 52-acre urban garden with a high tree diversity, especially for commonly planted genera like oaks. We used Brooklyn Botanic Garden’s *Plant Information Records System* to compile a list of focal oaks, compiling 15 native and 15 non-native oak trees (as this was the total number of non-native oaks). We surveyed the 15 native oak trees and 14 of the 15 non-native oak trees (one species’ leaves were inaccessible from the ground). For the native oaks, we focused on four species. These oaks were selected to include the two oaks (*Q. alba* and *Q. palustris*) common to our survey at Green-Wood Cemetery, and the two oaks (*Q. coccinea* and *Q. rubra*) that were the most common native species in the Garden (Supplemental Table 1).

The survey used the same methods as our work in Green-Wood Cemetery. Specifically, we measured gall abundance with a four minute survey walking around the entirety of the tree, and identified galls found to species. Herbivory measurements were not conducted due to low sample sizes for non-native species and varied leaf shapes among oak species.

#### Community data: iNaturalist analysis, Brooklyn

To examine galls at a broader spatial scale with sampling across a larger temporal scale, we analyzed data on galling arthropods across all of Brooklyn (Kings County) using records from iNaturalist, a community science database. These data were downloaded 17-Feb-2025, and span from 2017 to 2024, increasing the temporal coverage compared to the prior two substudies (Fig. 1, Supplemental Fig. 3). However, as iNaturalist is growing in popularity, we did not conduct any analyses across years to see if galling levels are variable (we instead focus on this in our herbarium analysis). We examined records for the three lineages of galling arthropods we observed at Green-Wood Cemetery and Brooklyn Botanic Garden (Cecidomyiidae family, Cynipini tribe, Eriophyidae family). Specifically, we used this to quantify the abundance and species richness of gall records for native and non-native trees. We used “Research Grade” data, which includes identifications that two thirds of users agree upon. To determine plant hosts, we used identifications by the observer when possible, and identified species ourselves if photos of the host plant were clear and easily identifiable. Records were excluded if hosts were only identified to genus or were not known. Once the host plant was identified, it was classified as native (from New York state), non-native (from Europe or Asia), or US native (from the US, but not from New York state), according to the USDA PLANTS database (Peterson et al. 1997).

#### Herbarium records: New York City

To determine if patterns of galling and herbivory held across an even broader spatial and temporal scales, we examined digitized herbarium specimen images from plants collected throughout New York City from 1883 to 2014 (Fig. 1, Supplemental Fig. 3). Images were obtained from the C. V. Starr Virtual Herbarium of the New York Botanical Garden on 21-Apr-2025 (Thiers et al. 2016). We focused our examinations on deciduous trees to match our Green-Wood Cemetery substudy, using species of *Acer*, *Prunus*, *Quercus*, and *Tilia* (Supplemental Table 2).

To analyze herbivory on herbarium sheets, we randomly selected 10 specimens from each congeneric species, using a random number generator to choose these specimens out of the total available. For some of the less frequently collected species, these 10 randomly selected specimens comprised the majority of the available collection. For *Quercus*, we used only *Q. alba* as our focal native and *Q. cerris* as our focal non-native (instead of *Q. robur* or *Q. acutissima*), as it was the only non-native oak with > 10 specimens. We then counted herbivory on 10 leaves per specimen. If there were greater than 10 leaves on a specimen, we counted the 10 most visible in a clockwise fashion from the bottom right (if leaves heavily overlapped, we only counted the top, visible leaf). We excluded specimens that had no leaves or were seedlings. Last, we averaged leaf percentage herbivory for the 10 leaves of each specimen using the same method as described above for Green-Wood Cemetery.

To analyze galls on herbarium sheets, we looked at all available trees of the same paired species of *Acer*, *Prunus*, *Quercus*, and *Tilia*, as well as all other identified species of *Quercus* in the database, matching our survey of Brooklyn Botanic Garden (Supplemental Table 3). Galls were measured exhaustively, with every leaf studied, recording the total number of galls made by each galling arthropod species per herbarium specimen image.

### Analytics

All code and data is available as GitHub repository at: https://github.com/liamengel/NYCNativeTrees-Arthropods.

For all analyses, we used R (The R Foundation, n.d.) with ggplot2 (Wickham 2016) for visualization. To compare gall abundance and mean percent herbivory within congeneric tree pairs at Green-Wood Cemetery and for the New York Botanical Garden herbarium specimens, we used Wilcoxon ranked-sum tests. To analyze gall abundance differences across our four oak species at Green-Wood Cemetery, we used a Kruskal-Wallis test. Next, due to the low sample size of galled non-native plants, we used Fisher’s exact test to compare iNaturalist and herbarium data gall abundance by origin. In our herbarium study, we used a negative binomial generalized linear model (GLM) with a log link function to examine gall abundance across time, testing year, origin, and their interaction. This type of GLM was used because it accounts for the many zeros and high variability in the data. Also in our herbarium study, we conducted an analysis of co-variance (ANCOVA) when examining mean percent herbivory across time to compare year and origin, and the interaction between these terms; this was run as “lm” and evaluated as “anova”.

For the analysis of herbivory in herbarium specimens, we first inspected the data for normality and heteroskedasticity, as we noticed that the *Prunus* data seemed to be biased compared to the other tree genera. In particular, we noticed that the *Prunus serotina* collection was highly biased towards early season collection dates (67% of specimens with dates were collected in early season, primarily May with one from June), while *Prunus serrulata* had most collections from late summer to fall (75% of specimens with dates were collected in August or later). Additionally, there was a bifurcation of sampling years with *Prunus*, with all but one *P. serotina* collected in 1985 or later, while every *P. serrulata* was collected between 1904 and 1945. When we inspected the qqnorm and residual plots, we found that including *Prunus* greatly skewed the results compared to plots that excluded *Prunus*. We also systematically inspected the qqnorm and residual plots for all other genera, and did not see the same skew. We therefore decided to exclude *Prunus* data from the analysis and plotting of herbivory in herbarium specimens and the ANCOVA. The galling analysis of *Prunus* from the herbarium samples included 49 specimens (compared to the 20 for herbivory that were randomly selected from the 49 total) and did not show the same bias in seasonality, and thus *Prunus* were not excluded from the galling timescale plot. For ease of viewing, some plots for herbivory and gall data were log-transformed and points were jittered.

Lastly, because there may be bias in historical collections due to changes in the types of specimens collected for herbaria, such as older collections potentially focusing on less-damaged leaves, we plotted a native-corrected interaction metric. A native-corrected interaction metric was calculated by setting the rate of herbivory on native plants as a baseline (zero), and calculating the relative herbivory on non-native plants as the interaction on non-native species minus that of natives collected during the same time period. We believe this will help reduce bias, as biases likely impact both native and non-native collections similarly. We binned data into 40-year blocks for calculations, starting with the years 1880, 1920, 1960, and 2000. For the years blocked from 2000, we could only use part of the block (until 2014), as we ran into the collection endpoint. While this is a much shorter block, it had the largest number of samples compared to other blocks, so we believe it is reasonable to keep as is.

## Results

### Overview

Overall, our New York City native tree species had more interactions with arthropods across multiple spatial and temporal scales, but with notable differences over time. As hypothesized, this was especially true for galls, which had clear and consistent differentiation between native and non-native trees. Herbivory was also higher in aggregate for native tree species, but was less consistent and usually not significantly different when comparing congeners. Specific results are broken down by substudy within sections about specialist and generalist interactions (galls and herbivory, respectively). Further examination of patterns across temporal and spatial scales is reserved for the discussion section.

### Specialist interactions: galls

We studied galling in all four substudies (Fig. 1). Across all temporal and spatial scales, gall-forming arthropod species richness was greater on native broadleaf deciduous trees, supporting our hypotheses of greater gall abundance on native tree species across spatial and temporal scales (*H*_*1*_, *H*_*3*_, *H*_*5*_). However, for certain genera we found no galls at all. Native oaks had the greatest richness of gall-forming arthropods across substudies. *Tilia* had abundant galls of a single species on both native and non-native trees. *Tilia cordata* was the only non-native congeneric paired species we observed to have any galls.

#### Natural experiment: Green-Wood Cemetery, Brooklyn

**Fig. 2:**
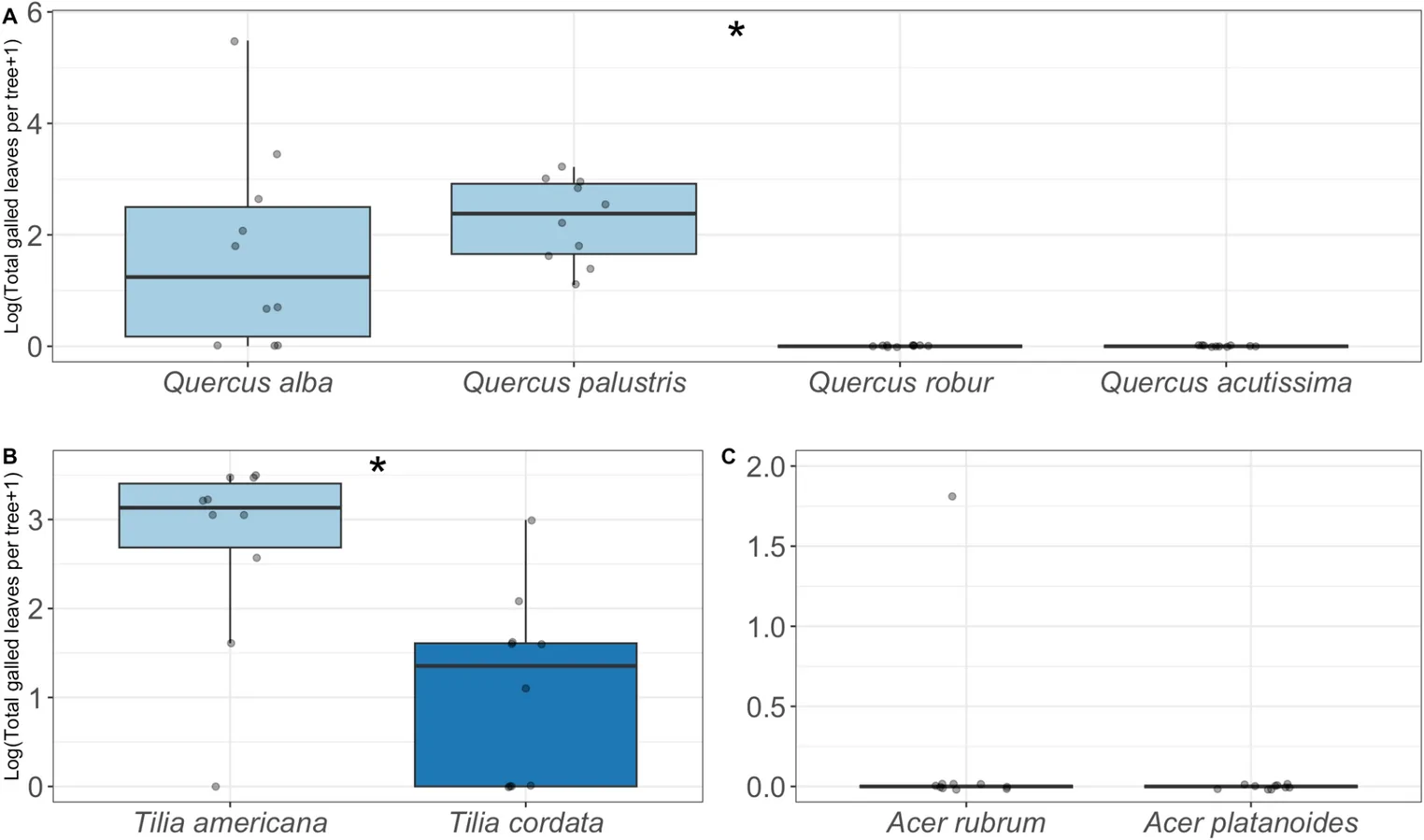
Box and whisker plot comparing the log-transformed number of galls per tree in native and non-native congeneric tree species pairs in Green-Wood Cemetery. Native trees are shown in light blue; non-native trees are shown in dark blue. For each of ten trees per species, galls observed were either summed in terms of presence/absence across 100 leaves (*Tilia*) or counted in four minutes of searching (all other genera). The midline of the box is the median, the upper and the lower portions of the box are the first and third quartiles, whiskers show data within 1.5x the interquartile range, and the dots represent outliers. The asterisks in plots **A** and **B** indicate statistically significant differences between native and non-native species. Results for *Prunus*, *Pinus*, and *Picea* are not plotted as they were invariant in our survey; all had no galls found

When comparing galls on all native and non-native trees surveyed at Green-Wood Cemetery, we found significantly greater gall abundance on native trees than non-natives (Wilcoxon test, W = 3222.5, *p* < 0.001), supporting our hypothesis of greater galling on native species (*H*_*1*_) (Fig. 2, Supplemental Table 4). This analysis included all genera, regardless of whether any galls were found. Galls were found on most native broadleaf tree species and one non-native broadleaf tree species, but no galls were found on *Prunus* or the conifers *Pinus* and *Picea*.

**Fig. 3:**
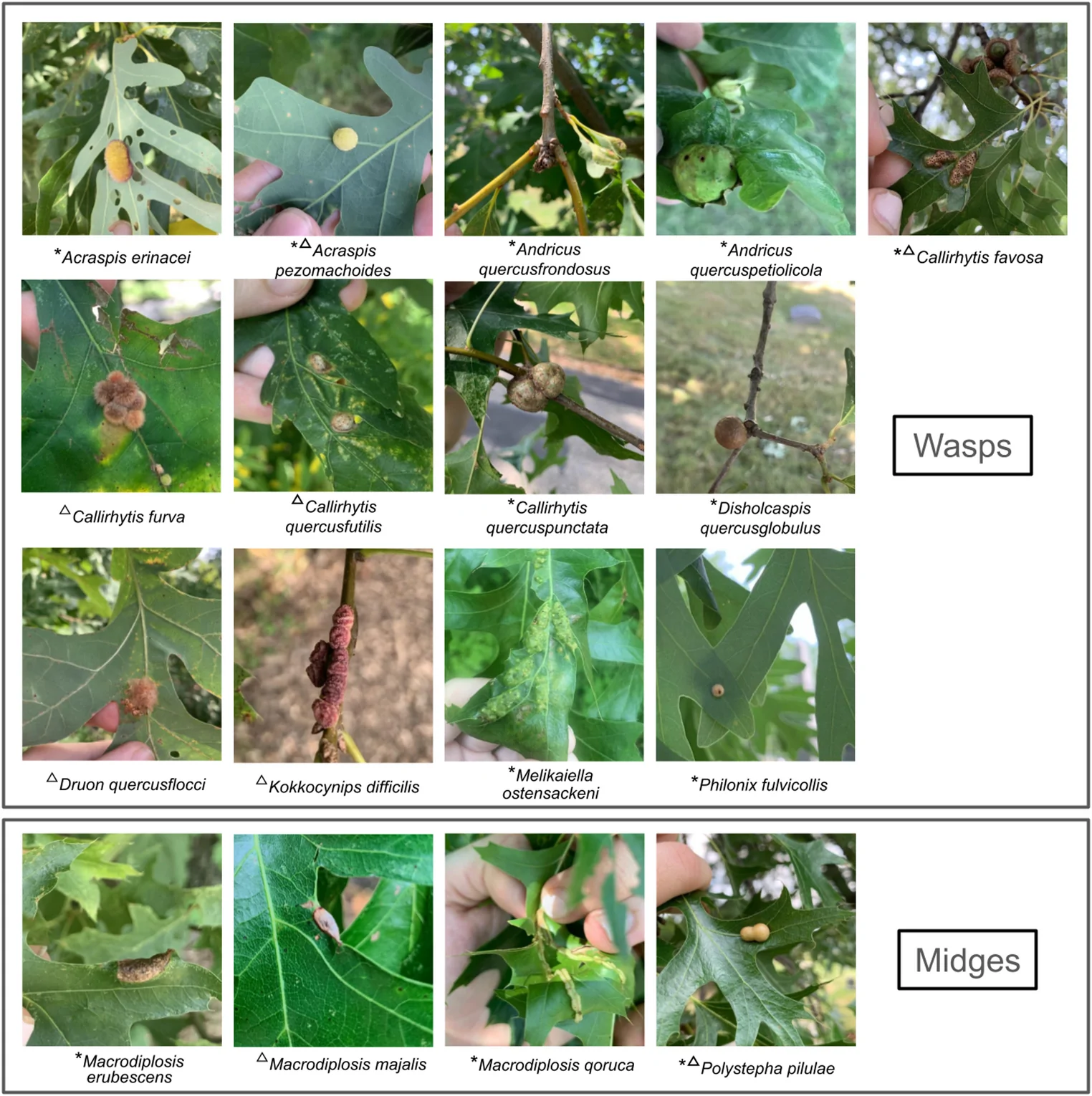
Galls found on native oak trees in Green-Wood Cemetery (*) and Brooklyn Botanic Garden (△), and presented in the order of Table 2 and Supplementary Table 4. The galls on the top two lines are formed by wasp species, the four galls on the third line are formed by midges. No galls were found on non-native species

We then examined native and non-native tree congeners that had galls to determine if there were significant differences. That is to say, we did not do congeneric comparisons of genera that did not have galls, as they were equivalent and invariant. Native oak trees were particularly species-rich for gall-forming arthropods; non-native oak trees had no galls (Figs. 2 and 3). A total of 14 species of gall-forming arthropods were found across the native trees sampled. Supplemental Table 4 details the exact species and their abundances on specific host trees. *Quercus palustris* had seven species of gall-formers; the most in this substudy. *Quercus alba* had the second-highest diversity of gall-formers, with six species observed. A non-parametric Kruskal-Wallis test for the four oak species examined found significant differences in galling (*p* < 0.001). Comparing individual oaks with Wilcoxon tests, we found native *Quercus alba* had significantly greater gall abundance than the non-native *Q. robur* (Fig. 2A; W = 85, *p* = 0.002), and native *Q. palustris* had significantly greater gall abundance than the non-native *Q. acutissima* (Fig. 2A; W = 100, *p* < 0.001).

Both native and non-native *Tilia* species had the same abundant gall-forming arthropod species, but the native *T. americana* had significantly more galls (Fig. 2B; W = 87.5, *p* = 0.005). No galls were detected on *Prunus*. We observed one individual of native *A. rubrum* with galls; we did not observe galls on any non-native *Acer platanoides* (Fig. 2C).

#### Diverse oak comparisons: Brooklyn Botanic Garden, Brooklyn

The survey of oak galls at Brooklyn Botanic Garden found the same pattern as our first substudy: native oaks had galls present, whereas non-native oaks had none (*H*_*1*_) (Supplemental Fig. 1; Wilcoxon test, W = 189, *p* < 0.001). In our survey, 12 of 15 native oak trees had galls (Table 2; Fig. 3).

**Table 2:** Counts of galls formed by arthropod species present on native oak species at Brooklyn Botanic Garden. No galls were found on non-native oak species

Group	Gall-forming species	Native *Quercus* (oak) species			
		*alba*	*coccinea*	*palustris*	*rubra*
Wasps	*Acraspis pezomachoides*	1			
	*Callirhytis favosa*			3	
	Callirhytis furva*		10	265	
	*Callirhytis quercusfutilis*	5			
	*Druon quercusflocci*	3			
	*Kokkocynips difficilis*			2	
Midges	*Macrodiplosis majalis*			2	
	*Polystepha pilulae*	2	2	105	63

*Callirhytis furva counts are of galled leaves, whereas other taxa are total gall counts per tree. This was done due to the far higher frequency of galling per leaf for this species

Eight species of galls were observed in Brooklyn Botanic Garden (Table 2). The two most abundant species were the wasp *Callihrytis furva* and the midge *Polystepha pilulae*, which were both found on multiple oak species. We also observed a newly recorded gall-forming arthropod species for New York, of the wasp *Kokkocynips difficilis* on *Q. palustris* (Nieves-Aldrey et al. 2021; Supplemental Fig. 2). This is the northernmost sighting of *Kokkocynips difficilis* on iNaturalist, and the first in New York.

#### Community data: iNaturalist analysis, Brooklyn

For iNaturalist records in Brooklyn, we found 700 total gall observations that were on identifiably native or non-native trees (this count does not include three *Quercus* species that hosted 22 total galls; these oaks are US native but two are at their range edge in NY). Of these, 98.4% (689 observations) were on native tree species (distributed among 42 tree species), whereas only 1.6% (11 observations) were found on non-native trees (distributed among four species).

Native plants hosted significantly more galls than non-native plants (Fisher’s exact test, *p* < 0.001; Table 3), supporting our hypothesis that results would be consistent across spatial scales (*H*_*3*_). Of the gall observations found on non-native hosts, 11 were on European and Asian host species (primarily non-native *Tilia*, with two records on *Buxus*). Additionally, 22 gall observations were on American oak species that are not native to NY. These oaks are mostly at their range edge on the east coast (*Quercus michauxii* and *Quercus shumardii* are both native to adjacent states), with one from the Mississippi River floodplain (*Quercus texana*; Stein et al. 2001) (Supplemental Table 5). Lastly, five galls that were found on hybridized *Quercus* specimens that were a part of a Cornell University oak hybridization program we counted as native, as one of the parental oak species was native, *Quercus bicolor* (Denig et al. 2014).

**Table 3:** Galling arthropods analyzed from iNaturalist data (examined February 17, 2025) focusing on the origin of the host plant they were found on (i.e., native or non-native). For this, we do not include the three oak species listed as US native (US species not native to New York state), as two were from adjacent states and thus less clear on how to categorize them

Group	Tribe/Family	Host plant origin	Total galls
Wasps	Cynipini	Native	503
		Non-native	0
Mites	Eriophyidae	Native	99
		Non-native	9
Midges	Cecidomyiidae	Native	87
		Non-native	2

#### Herbarium records: New York City

We analyzed 392 total herbarium specimens – 261 native tree specimens, and 131 non-native tree specimens. Of these 392 total specimens, only 23 had galls present; the vast majority of specimens (94%) did not have galls present. These specimens were collected across all of New York City over a 131 year period. Although these data are not standardized across space and time, utilizing herbarium specimens to study herbivory and galling has been performed in several previous studies (Ivison et al. 2023; Meineke et al. 2019).

Of the specimens that did support galling arthropods, native tree leaves had significantly more galls than non-native tree leaves (Fisher’s Exact Test, *p* = 0.001; Table 4). 22 of the 23 galled specimens were on native trees, and these were collected throughout the 131-year time period we considered (our analysis started in 1883, and the first galled specimen was from 1896). There was only a single galled non-native specimen, on leaves of the species *Tilia cordata*, which had three galls and was collected in 2009 (Supplemental Table 6). This matches our observations at Green-Wood Cemetery and Brooklyn Botanic Garden, where non-native trees (except *Tilia*) were not galled, and supports our spatial scale hypothesis (*H*_*3*_).

**Table 4:** Count of total galls and percent of specimens galled on native and non-native tree species within digitized herbarium specimens from the New York Botanical Garden. A total of 392 digitized herbarium samples were reviewed for this comparison, of which 23 specimens were found to have galls

Genus	Native			Non-native		
	Percent galled	Galling species	Total galls	Percent galled	Galling species	Total galls
*Acer*	6.67	1	46	0	0	0
*Prunus*	0	0	0	0	0	0
*Quercus*	8.02	12	66	0	0	0
*Tilia*	17.65	1	7	8.33	1	3

**Fig. 4:**
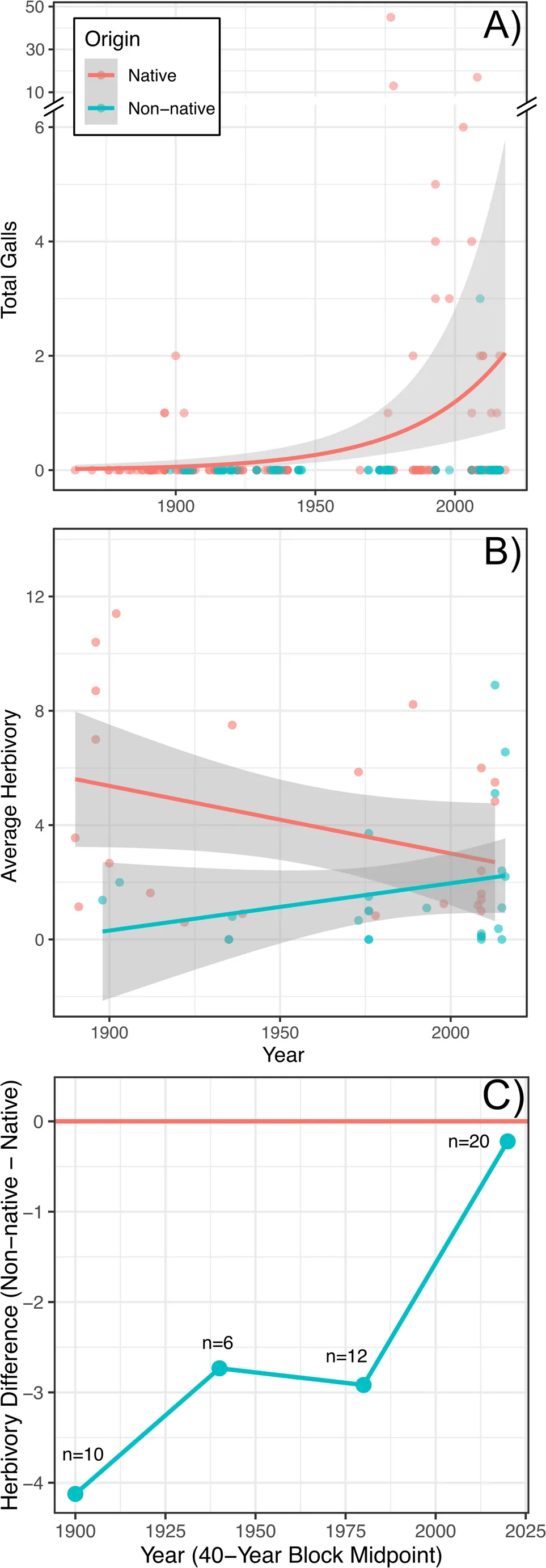
Plot of total galls and average herbivory on herbarium specimens over time, separated by origin (native vs. non-native species) using digitized herbarium specimens from the NYBG C. V. Starr Virtual Herbarium from 1831–2014. For the herbivory plot only (**B**), we excluded *Prunus* data as the samples were historically biased to certain seasons. Note the axis break in (**A**) along the count of total galls to account for three outlier specimens. For the galling plot (A), the negative binomial regression line was created for the native origin, with a 95% confidence interval shaded in gray. For the herbivory plot (**B**), a linear regression line was created for each origin, with 95% confidence intervals shaded in gray. Herbivory averaged for leaves of ten specimens for each species. Native and non-native tree species are listed in Supplemental Table 6. For the native-corrected interaction metric (**C**) we plot average percent herbivory difference between non-native and native specimens, binned into 40-year periods (midpoints plotted). For each period, average herbivory was calculated separately for native and non-native specimens, and the native average was set as a baseline (red horizontal line at zero). Points show the non-native average herbivory relative to this native baseline. Points falling below the baseline indicate lower average herbivory on non-native trees relative to native trees in that period. Sample sizes (native and non-native specimens combined) for each period are noted above each point

When looking across our 131-year timescale (1883–2014; Fig. 4A), we found that galling on native tree leaves increased significantly over time, (β = 0.03 ± 0.007, *p* < 0.001); corresponding to a 3% annual increase in gall abundance. There was no significant change in galling on non-natives (as only one non-native leaf had any galls), corresponding to no significant interaction between galling and origin (*p* = 0.347), and supporting our hypothesis of no change in galling for non-native species over time (*H*_*5*_).

### Generalist interactions: herbivory

We studied herbivory in two substudies: in Green-Wood Cemetery and in New York Botanical Garden herbarium records. Across both spatial and temporal scales considered, we found the same or slightly more herbivory on native trees than non-native congeneric trees (*H*_*2*_, *H*_*3*_), and found increased herbivory over time on non-native trees (*H*_*4*_). The only exception was for *Prunus* in herbarium records, where there was greater herbivory on non-native compared to native trees; however, there was an associated seasonal bias that collections were taken, as discussed in the Methods.

#### Natural experiment: Green-Wood Cemetery, Brooklyn

We only observed herbivory on broadleaf deciduous trees. No clear or easy to quantify herbivory was detected on conifers (*Pinus* or *Picea*), so there is no further discussion of conifers in these results.

**Fig. 5:**
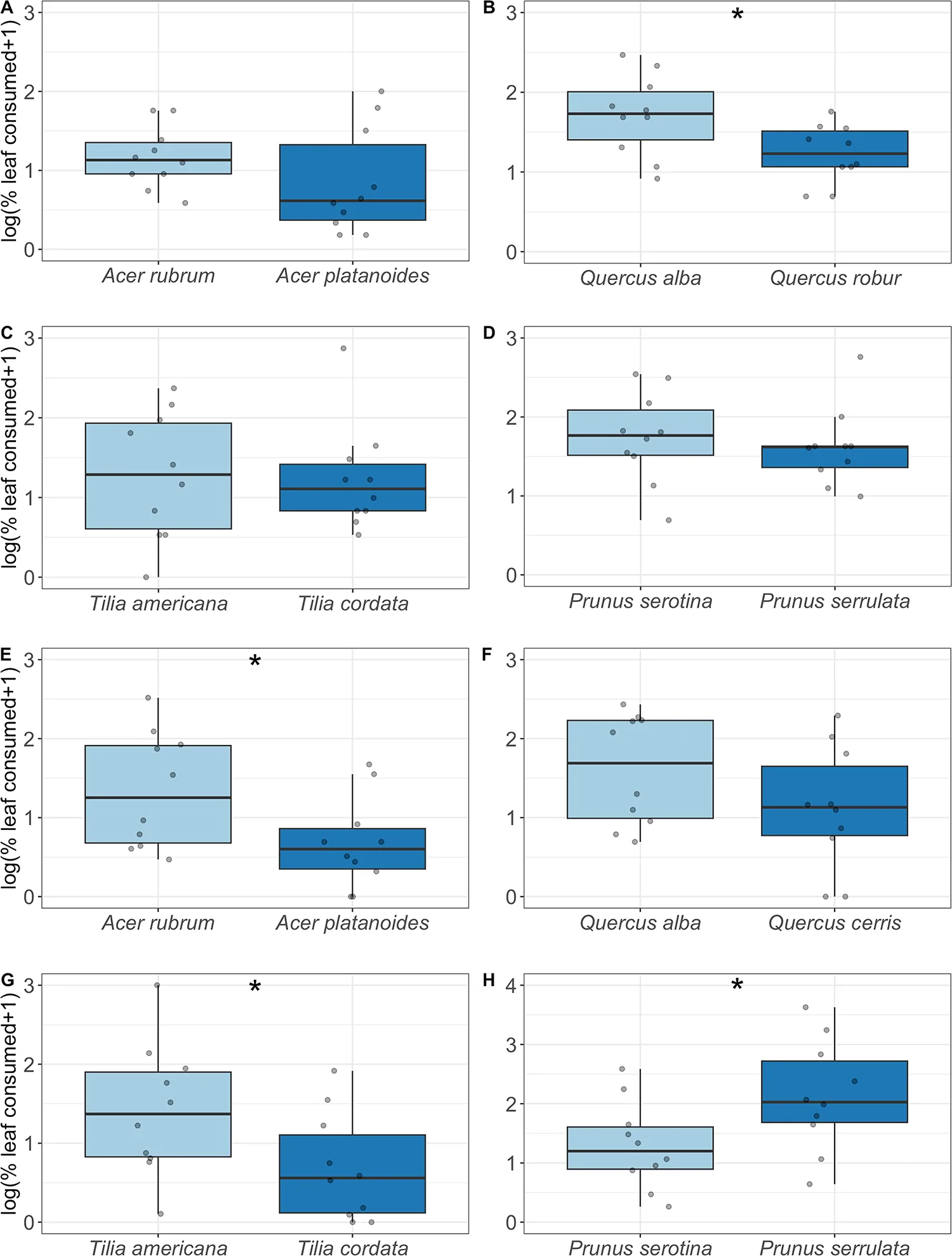
Box and whisker plots comparing the average percent herbivory per tree (measured as percent leaf consumed, averaged across 10 leaves per tree) on native and non-native congeneric tree species pairs, both for live trees in Green-Wood Cemetery (**A–D**) and herbarium specimens from New York Botanical Garden (**E–H**). Native trees are shown in light blue; non-native trees are shown in dark blue. Each dot represents the average for 10 different trees examined per species at Green-Wood and for NYBG specimens. Within each subplot, the midline of the box is the median, the upper and the lower portions of the box are the first and third quartiles, the whiskers show data within 1.5x the interquartile range, and the dots represent outliers. The asterisks in plots **A, B, E, G**, and **H** indicate statistically significant differences between native and non-native species

**Fig. 6:**
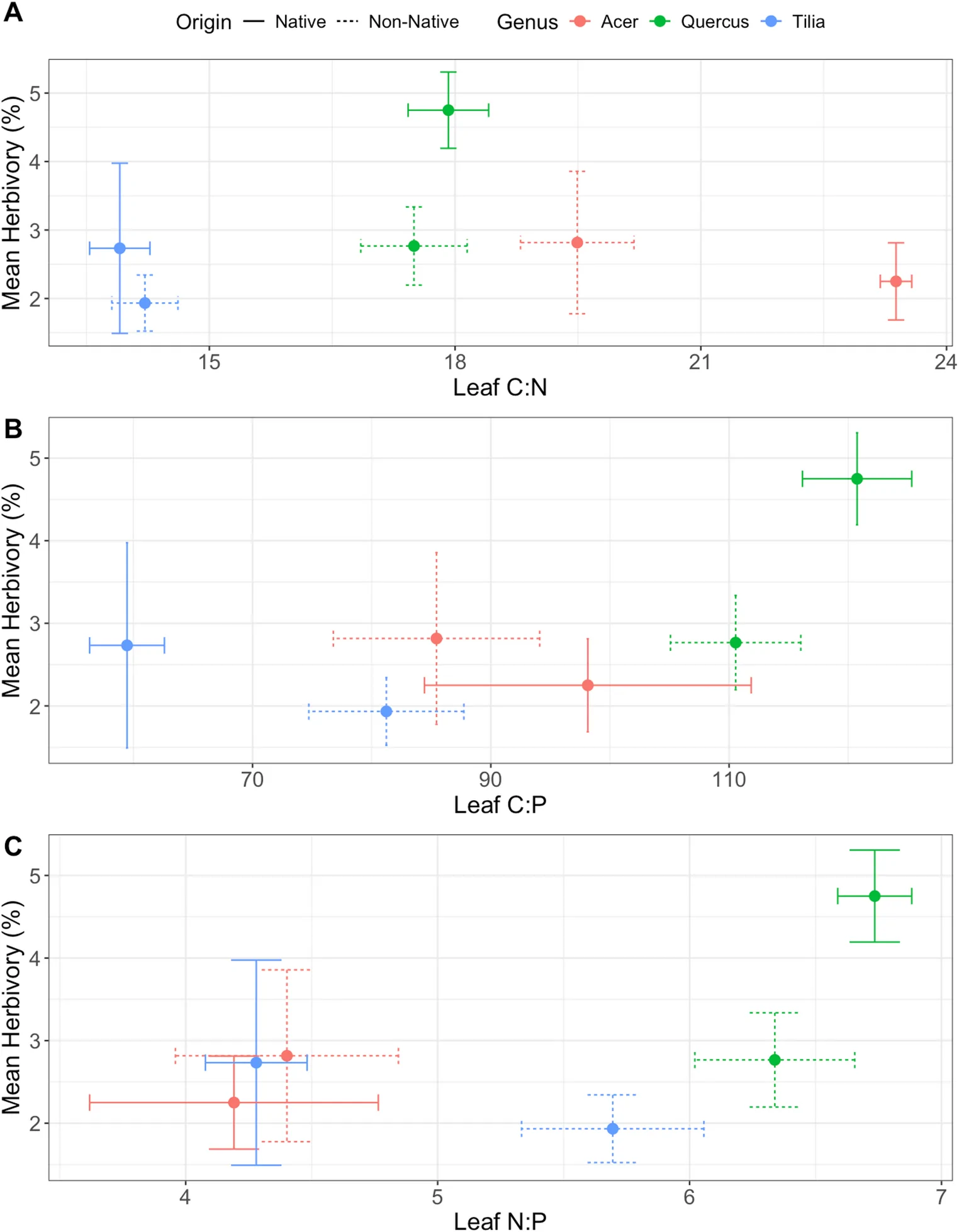
Leaf herbivory by leaf stoichiometry. Data gathered in 2024 in Green-Wood cemetery. Leaf herbivory and chemistry were measured in the same set of trees. Points show the mean values, and error bars show the standard error. Note that *Prunus* were not plotted because its leaf chemistry was not measured, and *Pinus* and *Picea* were not plotted because no herbivory was observed

Mean percent herbivory trended slightly higher for native trees on all broadleaf deciduous pairs (*Acer*, *Quercus*, *Tilia*, and *Prunus*) (Fig. 5). However, this difference was only statistically significant for *Quercus* (W = 78, *p* = 0.037) (Fig. 5), lending partial support to *H*_*1*_. Mean percent herbivory did not significantly differ between native and non-native congeners of *Acer* (W = 31.5, *p* = 0.173), *Tilia* (W = 51.5, *p* = 0.940) or *Prunus* (W = 61, *p* = 0.427) (Fig. 5A, C and D). Elemental C:N:P composition of leaves (baseline stoichiometry) did not correlate with leaf herbivory (Fig. 6), or with galling.

#### Herbarium records: New York City

Like in Green-Wood Cemetery, we looked at broadleaf deciduous trees. Within these genera (*Acer*, *Quercus*, *Tilia*, and *Prunus*), three out of four native trees had either the same or more herbivory than their non-native pairs (*H*_*1*_, *H*_*2*_, *H*_*3*_; Fig. 5). Both native *Acer* and *Tilia* had significantly greater herbivory than their non-native congeners (Fig. 5E and G; for both, Wilcoxon test, *Acer* W = 23, *p* = 0.045; *Tilia* W = 78.5, *p* = 0.034). For herbivory on *Quercus*, native and non-native congeneric trees did not differ significantly (Fig. 5F; W = 66.5, *p* = 0.226). The opposite pattern was observed for herbivory on *Prunus*, where non-native trees had significantly greater herbivory than their native congeners (Fig. 5H; W = 23, *p* = 0.045).

Across our 131-year timescale (1883–2014; Fig. 4B and C), we found that year of collection, tree origin, and the interaction between terms were all significant or approaching significance (*p* = 0.086, 0.017, 0.048). Broadly, herbivory on native trees decreased through time (slope = -0.024), while herbivory on non-native tree leaves increased (slope = + 0.017) (adjusted *R*² = 0.1811) (Fig. 4B). The native-corrected interaction metric indicates that herbivory on non-native plants increased through time, and has nearly equalized with herbivory on natives since 2000 (Fig. 4C).

## Discussion

### Overview

Despite the variability in our geographic study area across space and time, we found repeated evidence that generally supports our hypotheses and predictions about generalists and specialists relating to the enemy release hypothesis. Galls (a specialist interaction) were generally uncommon but present on all native tree species in at least one substudy. The only congeneric non-native tree to have galls was *Tilia cordata*, and a few US native *Quercus* growing at their range edge had galls in iNaturalist data. Herbivory (a generalist interaction) was common and often even on congeneric native and non-native trees, but some native trees had significantly greater herbivory levels than non-natives. Looking at herbarium specimens, this near equivalence in herbivory levels appears to have only occurred recently. Our four substudies had various strengths, including controlled field sites (one checked for stoichiometry, where we found that herbivory and galling differences cannot be explained by variation in tree leaf chemistry); a diversity of native and non-native congeners within an ecologically-important genus; wide-ranging, community-checked science records; and professionally deposited herbarium records over > 100 years.

Our findings support work conducted in other arboreta (Meineke et al. 2019) and in common garden experiments (Burghardt and Tallamy 2013, 2015), and highlights that non-native trees, even if grown locally for centuries, support fewer arthropods, both for specialists (galls) and, to a lesser degree, generalists (herbivory). We suggest that there has been insufficient time for coevolution of new insect interactions with non-native plants (Brändle et al. 2008; Meijer et al. 2016). As non-native plants increasingly dominate landscapes (van Kleunen et al. 2015), both primary consumers that utilize a limited subset of native plant lineages (like phytophagous insects) and their predator insects markedly decrease in abundance and diversity (Litt et al. 2014). Further downstream effects are probable, including reductions to insectivorous bird populations in areas dominated by non-natives (Helden et al. 2012; Narango et al. 2018).

### Spatial scales

Herbivory and galling patterns were mirrored across spatial scales, from a local study site (Green-Wood Cemetery) to city-wide (herbarium records) (*H*_3_). Compared to non-native congeners, native trees had slightly more or equal herbivory, as in previous studies (Tallamy and Shropshire 2009; Burghardt and Tallamy 2013; Pearse and Hipp 2014; Harvey et al. 2015). The only exception to this was herbarium records of *Prunus*, although strong seasonal differences in leaf collection likely account for greater herbivory on non-native trees compared to natives. The relationship between host plants and their insect enemies was also tighter for specialists than generalists, supporting our hypothesis (*H*_2_). Although there is high heterogeneity in the urban environment that can affect species interactions (e.g. Engel et al. 2020), we found generalist and specialist interactions remained relatively consistent across varied sites. Our results support predictions of the enemy release hypothesis: the non-native trees examined had few specialist enemies, while generalist herbivory was more similar between native and non-native congeners (although herbivory on non-natives was still slightly lower than for native trees) (Williamson 1996; Meijer et al. 2016).

Galls (formed by specialist arthropods) were found on three of the four native, broadleaf genera (*Quercus*, *Acer*, and *Tilia*), and also on non-native *Tilia*. In all four substudies, native oaks (*Quercus*) had the most abundant and diverse gall species, whereas non-native oaks had no observable galls, despite their long local planting history (Prince’s Select Catalogue of Fruit & Ornamental Trees 1849). We did not find galls on three tree genera (*Prunus*, *Picea*, and *Pinus*), which suggests an issue of sample size, as these trees can host galls. However, including these genera in our overall comparison at Green-Wood Cemetery still showed native trees hosted significantly more galls than non-natives.

### Time scales

Here, we used New York Botanical Garden herbarium data, which offers a 131-year perspective on plant-insect interactions. We were surprised that galls on native congeners increased through time. A biological change, like increases in gall-forming arthropods in New York City, could have caused this; for example, we made a new record of *K. difficilis* in our fieldwork. Alternatively, increased galling through time could be due to early herbarium collectors preferentially choosing aesthetically-pleasing specimens, which would lead to an undercount of galled leaves that may have been seen as diseased (Kozlov et al. 2021). However, we did not see clear patterns of herbivory biases through time in our native-corrected interaction metric, as many chewed leaves existed throughout time in herbarium collections and total herbivory on natives in collections has decreased over time. Whether this applies to galls is less clear, but possible. In support of our hypothesis (*H*_5_), and in contrast with galls on native trees, galls on non-native trees remained near zero, which implies that specialist insects still do not utilize non-native trees as hosts, even after many generations.

Temporal patterns in herbivory did not match those for galling. Matching our hypothesis (*H*_4_), herbivory on native congeners decreased through time, whereas herbivory on non-native trees increased. This implies that after many generations, generalist insects utilize novel, non-native trees as a food source, unlike highly specialized gall-forming arthropods (Schulte et al. 2025). Our findings match previous herbarium studies, like by Meineke et al. (2019), who found that herbivory on US native species decreased in urban areas across more than a century. Two European studies were more mixed, with one finding herbivory was similar on native and non-native congeners in Norway over 195 years (Ivison et al. 2023), and another finding microherbivory was greater on native plants across time, with equilibration on native and non-native woody plants occurring over 180 years (Schulte et al. 2025). Although herbivory levels on native and non-native trees may be equilibrating, potential interactions between year and tree origin complicate interpretations of our findings. Future work should disentangle these interacting terms by explicitly studying herbivory levels on native and non-native trees in a larger sample of herbaria over more time. This would better demonstrate whether herbivores can adapt to using non-native trees with increased residence time.

Our findings add further support to predictions of the enemy release hypothesis, with non-native species escaping attack by their co-evolved specialists. Specialists rarely switch hosts (Warren et al. 2021), meaning non-native plants remain disconnected from local specialist insects even after long periods of co-existence (Strong et al. 1984; Yi et al. 2024). Across > 100 years of herbarium records, we found consistent release from gall-forming enemies on non-native trees; even though many of these trees have been grown in the US for centuries, there may be many centuries longer before this release is over. However, we suggest that generalist interactions (percent herbivory) may be running out of enemy release. Herbivory is only slightly higher (and only occasionally significant) in our contemporary sampling. Additionally, herbivory on non-natives seems to have increased over time in the herbarium records, recently reaching levels similar to natives. What remains less clear for herbivory is whether a similar number of enemies (and thus biodiversity) will be supported by native and non-native trees once similar amounts of leaves are consumed.

### Stoichiometry

Variability within leaf stoichiometry (C:N and C:P) was not linked to leaf herbivory in our study (Fig. 6). Adding to this, our prior study (Herstoff et al. 2025) found that leaf nutrients at one of our study sites — Green-Wood Cemetery — were similar for native and non-native trees from the same genus, and there were no patterns associated with tree origin. However, nutrient ratios may help explain the lack of galling found on the coniferous genera, as previous research has found greater nutrient levels in deciduous leaves than coniferous needles (Sardans et al. 2011). However, there are limits to these data, as we only considered leaf chemistry as it relates to the plant’s nutritional value. Future work should examine whether other chemical differences, like levels of secondary metabolites (e.g. tannins), differ between native and non-native plants in ways that could explain our observed differences in galling and leaf herbivory.

### Strengths and biases of multiple approaches

While each substudy offers incomplete support of the enemy release hypothesis for urban trees, our multiple substudies approach fills gaps in our understanding across time and space. The live tree surveys provided highly constrained growing conditions, but had limited spatial areas. In contrast, our community science and herbarium records expanded our geographic scope, but did not have even sampling (e.g. certain neighborhoods and parks were overrepresented) (Supplemental Fig. 3) and did not explicitly account for the abundance of natives and non-native trees. New York City now maintains a tree inventory, with a large number of both native and non-native trees documented (Nowak et al. 2018), which could account for host availability in future studies.

For the combination of substudies, biases exist. All substudies took place within New York City, which is a single major metropolitan area, with accompanying high levels of pollution, increased temperature, fragmented habitat, and species filtering (Grimm et al. 2008; Alberti et al. 2020). Because the unique environmental conditions of urban areas are shared (McKinney 2006), a future study comparing our findings in New York City to other large metropolitan areas would support the generalizability of our results. Certain biases were specific to the use of herbarium records. Records were unevenly distributed across locations, years, and seasons, and older records may have avoided collecting leaves seen as damaged, possibly limiting the collection of galled leaves or leaves with herbivory. However, these biases may be relatively consistent across collections (Kozlov et al. 2021). We believe our native-corrected interaction metric helped to account for bias, and showed that non-native herbivory increased over time. We also note that, while most focal non-native cultivated street trees were non-invasive, *Acer platanoides* is indeed considered invasive locally. We included this species anyway, as it is still very commonly locally cultivated (Nowak et al. 2018).

### Urban conservation implications

As urban populations grow and urban land consumption continues to rapidly expand (World Bank, 2024), planners are tasked with both promoting biodiversity and addressing climate change (e.g. Dutta et al. 2025). Species selection of planted urban trees will increasingly influence the conservation of diverse arthropods and higher trophic level species (Stemmelen et al. 2022). Our findings suggest that selecting native trees provides an opportunity to strengthen conservation efforts, as native trees consistently supported greater gall-forming arthropod diversity. In our study, native tree species historically supported greater herbivory; but, these differences appeared to narrow through time, and non-native trees have recently begun to equilibrate with natives in supporting herbivory. Future work should identify the herbivore species utilizing native and non-native trees, as similar leaf damage levels may not represent similar arthropod diversity. Overall, we propose that city planners, private land managers, and individuals can strongly influence galling arthropods through their plant selections. Nurseries and landscaping companies can also encourage clients to plant a diversity of native trees.

Though galls and herbivory are often perceived as nuisances by horticulturalists, they represent an important and understudied indicator of arthropod diversity. New York City shares the environmental conditions common to major metropolitan areas (Alberti et al. 2008; Grimm et al. 2008), which might indicate that our findings are more broadly applicable. Previous studies have found mixed effects of urbanization on galling abundance (Sumoski et al. 2009; Meyer et al. 2020; De Andrade and Pereira-Colavite 2024), while a study on herbivory over time found decreased herbivory in urban areas (Meineke et al. 2019). Future research should examine these patterns with contemporary sampling and herbaria specimens on a more global scale.

Supporting arthropods in urban plantings may offer other benefits, such as ecosystem services (Schowalter et al. 2018). For example, one study found that galling decreased their host trees’ volatile organic compound emissions (Noe and Niinemets 2020). As urban systems can simulate the biological effects of climate change (Youngsteadt et al. 2015), understanding how planted trees and their associated arthropods respond to novel conditions may inform strategies for mitigating future climatic shifts. With tree species increasingly found outside their historical ranges (e.g., iNaturalist oak records), learning how both range-expanding native and non-native trees support urban arthropods is increasingly relevant amid rapid environmental change.

## Conclusions

Although many of the non-native trees we examined here have been cultivated locally for centuries (*Prince’s Select Catalogue of Fruit & Ornamental Trees*, 1849), they still have significantly fewer interactions with local galling arthropods and somewhat lower herbivory than natives. Although non-native species can fulfill important ecosystem services in urban areas (Sjöman et al. 2016), our results suggest that the non-native species surveyed are not integrating into the local communities in terms of specialist galls. Herbivory does seem to be increasing and likely equilibrating over time, but our results do not shed light on how many herbivorous insect species are supported to achieve this level of herbivory.

Our findings generally support enemy release hypothesis predictions on specialist and generalist arthropods across a range of spatial and temporal scales. However, as we find notable changes over time, we believe that more temporal analyses are critical for future studies. As urbanization increases, with an associated increase in managed tree planting, our findings should help urban planners prioritize native trees to better promote urban arthropods.

## Supplementary Information

Below is the link to the electronic supplementary material.

Supplementary Material 1

## Data Availability

Code and data is available here: https://github.com/liamengel/NYCNativeTrees-Arthropods.
