# Supplementary material for: The impact of non-native trees on galling and herbivory in New York City across space and time
Source: Oecologia. 2026 Sep 4;208(10):127. doi: 10.1007/s00442-026-05951-0 (PMC13545130; doi:10.1007/s00442-026-05951-0)
Supplement: Supplementary file 1 [file 442_2026_5951_MOESM1_ESM.docx]

**The impact of non-native trees on galling and herbivory in New York City across space and time**

**Supplemental Figure 1**

Box and whisker plot comparing the log-transformed number of galls averaged for native and non-native species of oak trees in Brooklyn Botanic Garden. For each species, which varied in number of trees surveyed (see Supplemental Table 1), galls observed were summed after four minutes of searching. The midline of the box is the median, the upper and the lower portions of the box are the first and third quartiles, whiskers show data within 1.5x the interquartile range, and the dots represent outliers. The asterisk indicates a statistically significant difference between native and non-native species (Wilcoxon test, W = 189, *p* < 0.001).

**Supplemental Figure 2**

Galls of the wasp species *Kokkocynips difficilis*. A) gall of *K. difficilis* noted with an arrow displayed on a pin oak (*Quercus palustris*) at Brooklyn Botanic Garden. B) map of *K. difficilis* found on iNaturalist focusing on our Brooklyn Botanic Garden observation, which is circled. C) map of *K. difficilis* found on iNaturalist showing the full US range.

**Supplemental Figure 3**

Sampling locations across our four substudies, including live tree sampling in 1) Green-Wood Cemetery (green) and 2) Brooklyn Botanic Garden (pink); and digital image records in 3) Brooklyn (white; iNaturalist records), and 4) New York City (light blue; New York Botanical Garden herbarium specimens). The top panel shows the sampling locations of the first two substudies in closer detail, while the bottom panel demonstrates their relative position within New York City and the sampling locations of the third and fourth substudies.

| **Supplemental Table 1**  Oak species (*Quercus*) surveyed at Brooklyn Botanic Garden, NY. Trees are separated by origin (native, non-native), and n shows the number of trees surveyed per species. Species with an asterisk (*) were also sampled at Green-Wood Cemetery. Subgenus and section are based on a phylogenomic analysis (Hipp et al., 2020). | | | | | |
| --- | --- | --- | --- | --- | --- |
| ***Quercus* species,  common name** | **Subgenus** | **Section** | **n** | **Native or year introduced** | **Source** |
| *coccinea*,  scarlet oak | *Quercus* | *Lobatae* | 4 | Native |  |
| *palustris*,  pin oak * | *Quercus* | *Lobatae* | 4 | Native |  |
| *rubra*,  northern red oak | *Quercus* | *Lobatae* | 5 | Native |  |
| *alba*,  white oak * | *Quercus* | *Quercus* | 2 | Native |  |
| *acutissima*,  sawtooth oak * | *Cerris* | *Cerris* | 6 | 1920 | https://plants.usda.gov/DocumentLibrary/factsheet/pdf/fs_quac80.pdf |
| *castaneifolia*,  chestnut-leaved oak | *Cerris* | *Cerris* | 1 | 1957 | https://arboretum.harvard.edu/arnoldia-stories/insights-from-a-sole-survivor-quercus-castaneifolia/ |
| *cerris*,  Turkey oak | *Cerris* | *Cerris* | 1 | 1898 | https://sweetgum.nybg.org/science/vh/specimen-details/?irn=1721992 |
| *libani*,  Lebanon oak | *Cerris* | *Cerris* | 1 | Unknown |  |
| *myrsinifolia*, bamboo-leaf oak | *Cerris* | *Cyclobalanopsis* | 1 | 1878 | https://www.bartlett.com/blog/japanese-evergreen-oak/ |
| *robur*,  English oak * | *Quercus* | *Quercus* | 2 | 1600s | https://plants.ces.ncsu.edu/plants/quercus-robur/ |
| *serrata*,  Konara oak | *Quercus* | *Quercus* | 2 | 1893 | https://landscapeplants.oregonstate.edu/plants/quercus-glandulifera |

| **Supplemental Table 2**  New York City native and non-native tree specimens analyzed for herbivory from New York Botanical Garden digital herbarium. An n of 10 was selected to keep sample sizes even, given a low total availability of certain species.  **Prunus* was not used in downstream analyses as the samples were biased by month of collection, with the native specimens collected earlier in the growing season than the non-native specimens. | | | | |
| --- | --- | --- | --- | --- |
| **Genus** | **Native species** | **n** | **Non-native species** | **n** |
| Maple | *Acer rubrum*, red maple | 10 | *A. platanoides*, Norway maple | 10 |
| Cherry* | *Prunus serotina*, black cherry | 10 | *P. serrulata*, East Asian cherry | 10 |
| Oak | *Quercus alba*, white oak | 10 | *Q. cerris*, Turkey oak | 10 |
| Linden | *Tilia americana*, basswood | 10 | *T. cordata*, little leaf linden | 10 |

| **Supplemental Table 3**  New York City native and non-native tree specimens analyzed for galling from New York Botanical Garden digital herbarium. All currently digitized New York City specimens for these species were used. n indicates the number of available, digitized specimens. | | | | |
| --- | --- | --- | --- | --- |
| **Genus** | **Native species** | **n** | **Non-native species** | **n** |
| Maple | *Acer rubrum*, red maple | 35 | *A. platanoides*, Norway maple | 57 |
| Cherry | *Prunus serotina*, black cherry | 13 | *P. serrulata*, East Asian cherry | 36 |
| Oak | *Quercus alba*, white oak | 16 | *Q. acutissima*, sawtooth oak | 5 |
|  | *Quercus bicolor*, swamp white oak | 25 | *Q. cerris*, Turkey oak | 11 |
|  | *Quercus borealis*, northern red oak | 3 | *Q. dentata*, sweet oak | 5 |
|  | *Quercus coccinea*, scarlet oak | 5 | *Q. frainetto*, Hungarian oak | 1 |
|  | *Quercus ilicifolia*, bear oak | 2 | *Q. pagoda*, cherrybark oak | 1 |
|  | *Quercus imbricaria*, shingle oak | 3 | *Q. robur*, English oak | 2 |
|  | *Quercus macrocarpa*, burr oak | 6 | *Q. suber*, cork oak | 1 |
|  | *Quercus marilandica*, blackjack oak | 10 |  |  |
|  | *Quercus michauxii*, swamp chestnut oak | 5 |  |  |
|  | *Quercus montana*, chestnut oak | 4 |  |  |
|  | *Quercus nigra*, water oak | 1 |  |  |
|  | *Quercus palustris*, pin oak | 21 |  |  |
|  | *Quercus phellos*, willow oak | 8 |  |  |
|  | *Quercus rubra*, northern red oak | 27 |  |  |
|  | *Quercus stellata*, post oak | 3 |  |  |
|  | *Quercus velutina*, black oak | 19 |  |  |
|  | *Quercus* x *brittoni*, Britton’s oak | 4 |  |  |
|  | *Quercus* x *hawkinsii,* Hawkins’ oak | 1 |  |  |
|  | *Quercus* x *heterophylla*, Bartram oak | 24 |  |  |
|  | *Quercus* x *leana*, lea oak | 1 |  |  |
|  | *Quercus* x *rudkinii*, Rudkin’s oak | 7 |  |  |
| Linden | *Tilia americana*, basswood | 17 | *T. cordata*, little leaf linden | 12 |

| **Supplemental Table 4**  Counts of galls formed by arthropod species compared for native and non-native tree species at Green-Wood Cemetery. Columns of trees are grouped as native and non-natives in the same genus for comparisons. | | | | | | | | | |
| --- | --- | --- | --- | --- | --- | --- | --- | --- | --- |
| Group | Gall-forming species | *Quercus* (oaks) | | | | *Acer* (maples) | | *Tilia* (lindens) | |
|  |  | Native | | Non-native | | Native | Non-native | Native | Non-native |
|  |  | *alba* | *palustris* | *robur* | *acutissima* | *rubrum* | *platanoides* | *americana* | *cordata* |
| Wasps | *Acraspis erinacei* | 256 |  |  |  |  |  |  |  |
|  | *Acraspis pezomachoides* | 2 |  |  |  |  |  |  |  |
|  | *Andricus quercusfrondosus* | 7 |  |  |  |  |  |  |  |
|  | *Andricus quercuspetiolicola* | 29 |  |  |  |  |  |  |  |
|  | *Callirhytis favosa* |  | 2 |  |  |  |  |  |  |
|  | *Callirhytis quercuspunctata* |  | 5 |  |  |  |  |  |  |
|  | *Disholcaspis quercusglobulus* | 2 |  |  |  |  |  |  |  |
|  | *Melikaiella ostensackeni* |  | 4 |  |  |  |  |  |  |
|  | *Philonix fulvicollis* | 2 |  |  |  |  |  |  |  |
| Mites | *Eriophyes tiliae* |  |  |  |  |  |  | 198* | 40* |
|  | *Vasates aceriscrumena* |  |  |  |  | 5 |  |  |  |
| Midges | *Macrodiplosis erubescens* |  | 32 |  |  |  |  |  |  |
|  | *Macrodisplosis qoruca* |  | 12 |  |  |  |  |  |  |
|  | *Polystepha pilulae* |  | 56 |  |  |  |  |  |  |
| **Tilia* counts are of galled leaves, whereas other taxa are total gall counts. This was done due to the far higher frequency of galling on *Tilia*. | | | | | | | | | |

| **Supplemental Table 5**  Counts of galls on host plants listed by arthropod taxa and species. Data obtained from iNaturalist for Brooklyn, NY, from the three galling taxa (family and tribe level) found in our Green-Wood Cemetery and Brooklyn Botanic Garden substudies. Within the Origin column, “Native” refers to New York state natives. “Non-native” refers to plants not from North America that were from Europe and Asia. “US native” refers to three *Quercus* that are from the US, but are outside of their range; note that *Quercus texana* is not found near New York state, but the other two US native oaks are native to adjacent states. | | | | |
| --- | --- | --- | --- | --- |
| **Taxa** | **Arthropod** | **Host Plant** | **Origin** | **Count** |
| Cecidomyiidae family | *Caryomyia aggregata* | *Carya glabra* | Native | 2 |
| Cecidomyiidae family | *Caryomyia ansericollum* | *Carya cordiformis* | Native | 8 |
| Cecidomyiidae family | *Caryomyia caryae* | *Carya glabra* | Native | 1 |
| Cecidomyiidae family | *Caryomyia caryae* | *Carya tomentosa* | Native | 1 |
| Cecidomyiidae family | *Caryomyia cynipsea* | *Carya tomentosa* | Native | 1 |
| Cecidomyiidae family | *Caryomyia eumaris* | *Carya tomentosa* | Native | 1 |
| Cecidomyiidae family | *Caryomyia persicoides* | *Carya glabra* | Native | 2 |
| Cecidomyiidae family | *Caryomyia persicoides* | *Carya tomentosa* | Native | 3 |
| Cecidomyiidae family | *Caryomyia purpurea* | *Carya glabra* | Native | 1 |
| Cecidomyiidae family | *Caryomyia sanguinolenta* | *Carya glabra* | Native | 2 |
| Cecidomyiidae family | *Caryomyia thompsoni* | *Carya glabra* | Native | 7 |
| Cecidomyiidae family | *Caryomyia thompsoni* | *Carya laciniosa* | Native | 1 |
| Cecidomyiidae family | *Caryomyia tuberculata* | *Carya glabra* | Native | 1 |
| Cecidomyiidae family | *Caryomyia tubicola* | *Carya glabra* | Native | 4 |
| Cecidomyiidae family | *Caryomyia tubicola* | *Carya tomentosa* | Native | 1 |
| Cecidomyiidae family | *Celticecis globosa* | *Celtis occidentalis* | Native | 1 |
| Cecidomyiidae family | *Celticecis spiniformis* | *Celtis occidentalis* | Native | 4 |
| Cecidomyiidae family | *Contarinia cerasiserotinae* | *Prunus serotina* | Native | 1 |
| Cecidomyiidae family | *Contarinia racemi* | *Prunus serotina* | Native | 2 |
| Cecidomyiidae family | *Dasineura pellex* | *Fraxinus pennsylvanica* | Native | 1 |
| Cecidomyiidae family | *Macrodiplosis erubescens* | *Quercus palustris* | Native | 1 |
| Cecidomyiidae family | *Macrodiplosis majalis* | *Quercus coccinea* | Native | 2 |
| Cecidomyiidae family | *Macrodiplosis majalis* | *Quercus palustris* | Native | 2 |
| Cecidomyiidae family | *Macrodiplosis majalis* | *Quercus texana* | US native | 1 |
| Cecidomyiidae family | *Macrodiplosis niveipila* | *Quercus rubra* | Native | 1 |
| Cecidomyiidae family | *Macrodiplosis qoruca* | *Quercus palustris* | Native | 1 |
| Cecidomyiidae family | *Monarthropalpus flavus* | *Buxus microphylla* | Non-native | 2 |
| Cecidomyiidae family | *Neolasioptera eupatorii* | *Ageratina altissima* | Native | 1 |
| Cecidomyiidae family | *Neolasioptera thurstoni* | *Helianthus divaricatus* | Native | 1 |
| Cecidomyiidae family | *Obolodiplosis robiniae* | *Robinia pseudoacacia* | Native | 2 |
| Cecidomyiidae family | *Polystepha globosa* | *Quercus rubra* | Native | 2 |
| Cecidomyiidae family | *Polystepha pilulae* | *Quercus marilandica* | Native | 1 |
| Cecidomyiidae family | *Polystepha pilulae* | *Quercus palustris* | Native | 13 |
| Cecidomyiidae family | *Polystepha pilulae* | *Quercus rubra* | Native | 1 |
| Cecidomyiidae family | *Polystepha pilulae* | *Quercus velutina* | Native | 1 |
| Cecidomyiidae family | *Polystepha pilulae* | *Quercus texana* | US native | 5 |
| Cecidomyiidae family | *Resseliella liriodendri* | *Liriodendron tulipifera* | Native | 5 |
| Cecidomyiidae family | *Taxodiomyia cupressiananassa* | *Taxodium distichum* | Native | 8 |
| Cynipini tribe | *Acraspis erinacei* | *Quercus alba* | Native | 49 |
| Cynipini tribe | *Acraspis pezomachoides* | *Quercus alba* | Native | 13 |
| Cynipini tribe | *Acraspis quercushirta* | *Quercus muehlenbergii* | Native | 1 |
| Cynipini tribe | *Amphibolips quercusostensackenii* | *Quercus rubra* | Native | 1 |
| Cynipini tribe | *Andricus capillatus* | *Quercus alba* | Native | 4 |
| Cynipini tribe | *Andricus chinquapin* | *Quercus bicolor* | Native | 2 |
| Cynipini tribe | *Andricus coronus* | *Quercus palustris* | Native | 1 |
| Cynipini tribe | *Andricus dimorphus* | *Quercus alba* | Native | 9 |
| Cynipini tribe | *Andricus dimorphus* | *Quercus macrocarpa* | Native | 1 |
| Cynipini tribe | *Andricus dimorphus* | *Quercus montana* | Native | 1 |
| Cynipini tribe | *Andricus dimorphus* | *Quercus michauxii* | US native | 1 |
| Cynipini tribe | *Andricus incertus* | *Quercus bicolor* | Native | 9 |
| Cynipini tribe | *Andricus nigricens* | *Quercus bicolor* | Native | 9 |
| Cynipini tribe | *Andricus pisiformis* | *Quercus alba* | Native | 3 |
| Cynipini tribe | *Andricus quercusfrondosus* | *Quercus alba* | Native | 2 |
| Cynipini tribe | *Andricus quercusfrondosus* | *Quercus bicolor* | Native | 14 |
| Cynipini tribe | *Andricus quercusfrondosus* | *Quercus stellata* | Native | 1 |
| Cynipini tribe | *Andricus quercuspetiolicola* | *Quercus alba* | Native | 2 |
| Cynipini tribe | *Andricus quercuspetiolicola* | *Quercus bicolor* | Native | 20 |
| Cynipini tribe | *Andricus quercuspetiolicola* | *Quercus marilandica* | Native | 1 |
| Cynipini tribe | *Andricus quercusstrobilanus* | *Quercus bicolor* | Native | 37 |
| Cynipini tribe | *Andricus quercusstrobilanus* | *Quercus bicolor Cornell hybrid* | Native | 1 |
| Cynipini tribe | *Andricus quercusutriculus* | *Quercus alba* | Native | 1 |
| Cynipini tribe | *Andricus weldi* | *Quercus alba* | Native | 5 |
| Cynipini tribe | *Callirhytis balanacea* | *Quercus palustris* | Native | 2 |
| Cynipini tribe | *Callirhytis balanacea* | *Quercus rubra* | Native | 1 |
| Cynipini tribe | *Callirhytis favosa* | *Quercus coccinea* | Native | 2 |
| Cynipini tribe | *Callirhytis favosa* | *Quercus palustris* | Native | 5 |
| Cynipini tribe | *Callirhytis furva* | *Quercus palustris* | Native | 11 |
| Cynipini tribe | *Callirhytis furva* | *Quercus texana* | Non-native | 2 |
| Cynipini tribe | *Callirhytis glandulus* | *Quercus alba* | Native | 1 |
| Cynipini tribe | *Callirhytis lanata* | *Quercus coccinea* | Native | 7 |
| Cynipini tribe | *Callirhytis lanata* | *Quercus rubra* | Native | 12 |
| Cynipini tribe | *Callirhytis lanata* | *Quercus shumardii* | US native | 1 |
| Cynipini tribe | *Callirhytis pigra* | *Quercus coccinea* | Native | 1 |
| Cynipini tribe | *Callirhytis piperoides* | *Quercus rubra* | Native | 1 |
| Cynipini tribe | *Callirhytis piperoides* | *Quercus x hawkinsiae* | Native | 1 |
| Cynipini tribe | *Callirhytis quercuscornigera* | *Quercus palustris* | Native | 25 |
| Cynipini tribe | *Callirhytis quercuscornigera* | *Quercus texana* | US native | 1 |
| Cynipini tribe | *Callirhytis quercusfutilis* | *Quercus alba* | Native | 2 |
| Cynipini tribe | *Callirhytis quercusgemmaria* | *Quercus rubra* | Native | 1 |
| Cynipini tribe | *Callirhytis quercusoperator* | *Quercus coccinea* | Native | 2 |
| Cynipini tribe | *Callirhytis quercusoperator* | *Quercus ilicifolia* | Native | 1 |
| Cynipini tribe | *Callirhytis quercusoperator* | *Quercus rubra* | Native | 6 |
| Cynipini tribe | *Callirhytis quercuspunctata* | *Quercus palustris* | Native | 4 |
| Cynipini tribe | *Callirhytis quercusventricosa* | *Quercus velutina* | Native | 2 |
| Cynipini tribe | *Callirhytis scitula* | *Quercus coccinea* | Native | 1 |
| Cynipini tribe | *Disholcaspis quercusglobulus* | *Quercus alba* | Native | 27 |
| Cynipini tribe | *Disholcaspis quercusglobulus* | *Quercus rubra* | Native | 2 |
| Cynipini tribe | *Disholcaspis quercusmamma* | *Quercus alba* | Native | 1 |
| Cynipini tribe | *Disholcaspis quercusmamma* | *Quercus bicolor* | Native | 33 |
| Cynipini tribe | *Disholcaspis quercusmamma* | *Quercus bicolor Cornell hybrid* | Native | 1 |
| Cynipini tribe | *Disholcaspis quercusmamma* | *Quercus macrocarpa* | Native | 1 |
| Cynipini tribe | *Druon ignotum* | *Quercus bicolor* | Native | 12 |
| Cynipini tribe | *Druon ignotum* | *Quercus bicolor Cornell hybrid* | Native | 1 |
| Cynipini tribe | *Druon quercusflocci* | *Quercus alba* | Native | 4 |
| Cynipini tribe | *Dryocosmus quercuslaurifoliae* | *Quercus phellos* | Native | 10 |
| Cynipini tribe | *Dryocosmus quercusnotha* | *Quercus palustris* | Native | 7 |
| Cynipini tribe | *Dryocosmus quercusnotha* | *Quercus phellos* | Native | 1 |
| Cynipini tribe | *Dryocosmus quercusnotha* | *Quercus texana* | US native | 2 |
| Cynipini tribe | *Dryocosmus quercuspalustris* | *Quercus imbricaria* | Native | 4 |
| Cynipini tribe | *Dryocosmus quercuspalustris* | *Quercus palustris* | Native | 9 |
| Cynipini tribe | *Dryocosmus quercuspalustris* | *Quercus rubra* | Native | 5 |
| Cynipini tribe | *Dryocosmus quercuspalustris* | *Quercus shumardii* | US native | 3 |
| Cynipini tribe | *Dryocosmus quercuspalustris* | *Quercus texana* | US native | 4 |
| Cynipini tribe | *Kokkocynips difficilis* | *Quercus palustris* | Native | 1 |
| Cynipini tribe | *Kokkocynips rileyi* | *Quercus coccinea* | Native | 4 |
| Cynipini tribe | *Kokkocynips rileyi* | *Quercus rubra* | Native | 4 |
| Cynipini tribe | *Melikaiella ostensackeni* | *Quercus palustris* | Native | 1 |
| Cynipini tribe | *Melikaiella ostensackeni* | *Quercus rubra* | Native | 3 |
| Cynipini tribe | *Melikaiella tumifica* | *Quercus coccinea* | Native | 5 |
| Cynipini tribe | *Melikaiella tumifica* | *Quercus rubra* | Native | 6 |
| Cynipini tribe | *Neuroterus floccosus* | *Quercus bicolor* | Native | 7 |
| Cynipini tribe | *Neuroterus floccosus* | *Quercus bicolor x aliena var. acutiserrata* | Native | 1 |
| Cynipini tribe | *Neuroterus quercusbatatus* | *Quercus alba* | Native | 1 |
| Cynipini tribe | *Neuroterus quercusbatatus* | *Quercus bicolor* | Native | 6 |
| Cynipini tribe | *Neuroterus quercusverrucarum* | *Quercus bicolor* | Native | 2 |
| Cynipini tribe | *Neuroterus tantulus* | *Quercus alba* | Native | 2 |
| Cynipini tribe | *Neuroterus umbilicatus* | *Quercus bicolor* | Native | 5 |
| Cynipini tribe | *Neuroterus umbilicatus* | *Quercus bicolor x aliena var. acutiserrata* | Native | 1 |
| Cynipini tribe | *Philonix fulvicollis* | *Quercus alba* | Native | 31 |
| Cynipini tribe | *Phylloteras poculum* | *Quercus alba* | Native | 22 |
| Cynipini tribe | *Zopheroteras guttatum* | *Quercus imbricaria* | Native | 1 |
| Cynipini tribe | *Zopheroteras guttatum* | *Quercus palustris* | Native | 1 |
| Cynipini tribe | *Zopheroteras guttatum* | *Quercus phellos* | Native | 2 |
| Cynipini tribe | *Zopheroteras guttatum* | *Quercus texana* | US native | 2 |
| Cynipini tribe | *Zopheroteras hubbardi* | *Quercus rubra* | Native | 4 |
| Eriophyidae family | *Aceria brachytarsa* | *Juglans nigra* | Native | 2 |
| Eriophyidae family | *Aceria caryae* | *Carya cordiformis* | Native | 2 |
| Eriophyidae family | *Aceria caryae* | *Carya ovata* | Native | 1 |
| Eriophyidae family | *Aceria caulis* | *Juglans nigra* | Native | 4 |
| Eriophyidae family | *Aceria celtis* | *Celtis occidentalis* | Native | 6 |
| Eriophyidae family | *Aceria cinereae* | *Juglans cinerea* | Native | 2 |
| Eriophyidae family | *Aceria dina* | *Nyssa sylvatica* | Native | 8 |
| Eriophyidae family | *Aceria fraxini* | *Fraxinus pennsylvanica* | Native | 1 |
| Eriophyidae family | *Aceria negundi* | *Acer negundo* | Native | 2 |
| Eriophyidae family | *Aceria nyssae* | *Nyssa sylvatica* | Native | 6 |
| Eriophyidae family | *Aceria parulmi* | *Ulmus americana* | Native | 3 |
| Eriophyidae family | *Aceria triplacis* | *Quercus alba* | Native | 1 |
| Eriophyidae family | *Aceria vaga* | *Carya cordiformis* | Native | 5 |
| Eriophyidae family | *Aculops aenigma* | *Salix interior* | Native | 1 |
| Eriophyidae family | *Aculops rhois* | *Rhus aromatica* | Native | 13 |
| Eriophyidae family | *Aculops rhois* | *Toxicodendron radicans* | Native | 4 |
| Eriophyidae family | *Aculus tetanothrix* | *Salix nigra* | Native | 1 |
| Eriophyidae family | *Colomerus vitis* | *Vitis 'Prairie Star'* | Native | 1 |
| Eriophyidae family | *Eriophyes cerasicrumena* | *Prunus serotina* | Native | 8 |
| Eriophyidae family | *Eriophyes leiosoma* | *Tilia americana* | Native | 1 |
| Eriophyidae family | *Eriophyes tiliae* | *Tilia americana* | Native | 15 |
| Eriophyidae family | *Eriophyes tiliae* | *Tilia cordata* | Non-native | 6 |
| Eriophyidae family | *Eriophyes tiliae* | *Tilia tomentosa* | Non-native | 1 |
| Eriophyidae family | *Eriophyes tiliae* | *Tilia x europaea* | Non-native | 2 |
| Eriophyidae family | *Vasates quadripedes* | *Acer saccharinum* | Native | 12 |

| **Supplemental Table 6**  Counts of galls formed by arthropod species on native and non-native tree species within digitized herbarium specimens from the New York Botanical Garden. The asterisk next to *Tilia cordata* indicates it is the only non-native tree species with galling. | | | | | | | | | | |
| --- | --- | --- | --- | --- | --- | --- | --- | --- | --- | --- |
| Group | Galling species | *Quercus* (oak) | | | | | | *Acer* (maple) | *Tilia* (linden) | |
|  |  | *alba* | *bicolor* | *macrocarpa* | *palustris* | *rubra* | *velutina* | *rubrum* | *americana* | *cordata** |
| Wasps | *Acraspis pezomachoides* | 12 |  |  |  |  |  |  |  |  |
|  | *Andricus sp.* |  | 2 |  |  |  |  |  |  |  |
|  | *Druon quercusflocci* | 1 |  |  | 4 |  |  |  |  |  |
|  | *Dryocosmus quercusnotha* |  |  |  |  |  | 1 |  |  |  |
|  | *Philonix fulvicollis* | 1 |  |  |  |  |  |  |  |  |
|  | *Phylloteras poculum* | 5 |  |  |  |  |  |  |  |  |
| Midges | *Acericecis ocellaris* |  |  |  |  |  |  | 46 |  |  |
|  | *Macrodiplosis erubescens* | 1 |  |  |  |  | 4 |  |  |  |
|  | *Macrodiplosis majalis* |  |  |  |  |  | 11 |  |  |  |
|  | *Polystepha globosa* |  |  |  |  | 5 | 9 |  |  |  |
|  | *Polystepha pilulae* |  |  |  | 6 | 4 |  |  |  |  |
| Mites | *Eriophyes tiliae* |  |  |  |  |  |  |  | 7 | 3 |
